# Bacterial Transformation of Adamantane and Its Derivatives: Regioselectivity and Biocatalytic Approaches

**DOI:** 10.3390/biology14101429

**Published:** 2025-10-17

**Authors:** Anastasia A. Ivanova, Anna A. Vetrova

**Affiliations:** Federal Research Center “Pushchino Scientific Center for Biological Research of the Russian Academy of Sciences”, 142290 Pushchino, Russia

**Keywords:** adamantane, bacterial transformation, hydrocarbons, biocatalysis

## Abstract

Adamantane is a unique tricyclic hydrocarbon with a rigid, diamond-like structure. It is widely distributed in natural hydrocarbons and has significant potential applications in medicine, materials science, and pharmaceuticals. Despite its high chemical stability, biocatalytic methods using cytochrome P450 enzymes and microorganisms, such as *Pseudomonas* strains and actinobacteria, demonstrate high regional specificity in the hydroxylation and modification of adamantane, primarily at tertiary C–H bonds. This review summarises the latest data on the mechanisms and pathways of the microbial transformation of adamantane and its derivatives, including the key metabolites and enzymatic systems involved. The advantages of biocatalysis—namely, high selectivity, environmental friendliness and mild reaction conditions—are highlighted as a promising approach to the synthesis of functional adamantane derivatives for use in the development of innovative drugs and materials. The limitations of current methods and prospects for development are also discussed, including searching for new microorganisms and regulating enzymatic activity to increase the efficiency of biotransformation.

## Simple Summary

Adamantane is a rare type of molecule found in oil that resembles a tiny, rigid cage made of carbon atoms. The material’s robust structure renders it a valuable component in modern medicines and materials. However, its inherent resistance to modification or breakdown by traditional chemical methods poses a significant challenge. This study explores how certain bacteria can assist scientists in making useful changes to adamantane in gentle, eco-friendly ways. The review summarises recent findings regarding the selective addition of oxygen to adamantane by bacteria, a process that results in the formation of novel substances with potential applications in the fields of drug development and advanced materials. The study identifies the most effective types of bacteria for effecting these changes and explores ways to enhance their performance. The findings indicate that bacterial methodologies exhibit superior specificity and environmental compatibility in comparison to conventional chemical processes. The enhancement of comprehension regarding these natural methodologies has the potential to facilitate the production of medicines and materials that would otherwise be challenging to create, thereby conferring benefits to healthcare, environmental safety and technology. This knowledge facilitates the development of innovative products inspired by nature.

## 1. Introduction

Diamond hydrocarbons are a class of organic compounds characterised by a rigidly fixed three-dimensional polycyclic structure corresponding to fragments of the diamond lattice. Diamondoids have been identified as components of crude oil and extracts of fossil coals and sedimentary rocks and are also present in the products of thermal decomposition of natural organic substances, such as source rocks and kerogen. Moreover, these representatives are present in various fractions of hydrocarbon mixtures, including saturated, aromatic, resinous, and asphaltene components. In these mixtures, they can act as structural markers and influence the physicochemical properties of natural systems [1].

The concentration of adamantane in oil exceeds the levels found in other petroleum diamondoids, thus making it the most common representative of this group of compounds [2]. Natural adamantane was first discovered in crude oil extracted from a field near the town of Godonín, Czechoslovakia, in 1933 [3,4,5]. Paul von Rage-Schleier first implemented the synthetic method in 1957 [6]. Notwithstanding this fact, the selective functionalisation of adamantane remains a challenging task due to its high thermal stability and unique structure.

Adamantane is a tricyclic hydrocarbon characterised by a rigid structure, consisting of cyclohexane rings in a chair conformation. The term ‘adamantane’ is derived from the Greek word διαμάντι (diamánti), which translates as ‘diamond’, on account of the chemical structure of the substance being analogous to a three-dimensional fragment of a diamond crystal lattice [2].

The structural and chemical properties of adamantane suggest its potential application in the creation of three-dimensional framework structures and drug delivery carriers. Adamantane functions as a molecular building block for two fundamentally different types of structures. Firstly, it can be used as a fragment with a tetrahedral carbon skeleton, providing the basis for the synthesis of covalently modifiable systems, such as adamantane dendrimers. Secondly, it can be used as a component of self-organising supramolecular assemblies, for example, as part of lipid vesicles or as a guest molecule in host-guest complexes with cyclodextrin, where it exhibits high specificity of interaction with the carrier [7].

Adamantane is a chemical compound that has found widespread application in the domain of medical chemistry, primarily due to its distinctive physicochemical properties. This structural element is characterised by its exceptional stability and resistance to oxidation. The primary benefits of this component in the context of specific drug delivery systems (DDSs) are outlined below [7]:

(1) The substance exhibits elevated chemical stability, encompassing a high degree of resistance to oxidation processes.
(2) Hydrophobic properties are exhibited, thereby ensuring stability in aqueous solutions.
(3) The compact molecule size facilitates integration into pharmaceutical compositions without significantly altering their physicochemical characteristics.
(4) It possesses lipophilicity, which plays a key role in ensuring effective penetration through lipid biological membranes.
(5) Its significant adsorption capacity on material surfaces has been demonstrated to enhance the applicability of these materials in drug delivery systems (DDSs) that require fixation on specific surfaces.
(6) The substance is characterised by low toxicity and an absence of allergic reactions, thus ensuring its biocompatibility.

However, it is these same properties that make chemical modification difficult, stimulating interest in biocatalytic strategies.

Adamantane derivatives have found practical application in the form of medicinal products exhibiting various biological activities [8,9]. The adamantyl radical influences hypoglycaemic, antitumour, immunosuppressive, antibacterial, antifungal, hormonal, analgesic, antipyretic, anti-inflammatory, hypotensive, choleretic, antiarrhythmic, sedative, neuroprotective, antimalarial and anticholinesterase activities. It also has a stimulating effect on the central nervous system [10]. Efficiently creating such biologically active derivatives requires methods that can ensure controlled and selective functionalisation. This is a challenging task for chemical modification due to the high dissociation energies of C–H bonds. This necessitates the development of biocatalytic approaches that can achieve high yields and selectivity.

The two best-known derivatives of adamantane are amantadine and memantine [11]. Memantine (marketed as Ebixa) has been approved for medical use in Canada since 2004 for the symptomatic treatment of patients with moderate to severe Alzheimer’s disease dementia. It has also been approved for this use in the United States and Australia since 2003 [12]. Substituted adamantanes are also used to treat other diseases. For example, adapalene [13] is an acne medication, and saxagliptin [14] is a dipeptidyl peptidase-4 (DPP-4) inhibitor used in the treatment of diabetes mellitus.

Thanks to significant advances in the synthesis and selective substitution of adamantane, it has become a readily available starting material, opening up a wide range of applications in various fields. As well as being used in medical chemistry, adamantane derivatives are widely used in the development of catalysts [15] and nanomaterials [16] thanks to their unique structural and biological properties. The synthesis of substituted adamantanes and higher substituted diamondoids often involves the use of carbocation or radical intermediates, which are more stable and reactive than simple hydrocarbon derivatives [9]. The direct substitution of adamantane molecules has long been used as a model system for developing methods of directly functionalising C–H bonds. This is particularly true given the unusually high bond dissociation energies (BDEs) of this class of compounds (96 and 99 kcal × mol^−1^ for secondary and tertiary C–H bonds, respectively) [17]. The main problem lies in achieving selectivity when functionalising the non-equivalent secondary and tertiary positions of adamantane, higher-molecular-weight diamondoids, and their substituted derivatives. The presence of several different types of C–H bonds can lead to the formation of complex mixtures of substitution products, which limits the complexity of reaction compounds.

Thanks to its high stability and low susceptibility to microbial degradation, adamantane can be used as a marker for microbiological and geochemical processes in oils and gas condensates [18]. At the same time, the ability of certain microorganisms to transform it creates opportunities for environmentally friendly biotechnologies and the biocatalytic synthesis of bioactive compounds. Microbial hydroxylation, which can be regio- and/or stereo-selective on the unactivated carbon atom of alicyclic and heterocyclic compounds, is an interesting and promising method for synthesising chemical substances. Biocatalytic methods, which are based on the ability of microbial enzymes to provide high selectivity and mild reaction conditions, can overcome the limitations of traditional chemical synthesis.

One of the first studies [19] reported that the cytochrome P450cam monooxygenase enzyme from the bacterium *Pseudomonas putida* selectively oxidises adamantane to form 1-adamantanol. However, the enzyme’s catalytic activity was very low.

The urgent task at present is to search for and study microorganisms capable of ensuring high selectivity in the production of adamantane derivatives, which play an important role in the development of functional materials and effective drugs. This biocatalytic approach could increase the regioselectivity of adamantane transformation, offering promise for medicine and materials science. A systematic analysis and integration of the current data on the microbial biotransformation of adamantanes is important for expanding our fundamental knowledge of the enzymatic mechanisms involved, as well as for their practical application in biotechnology, the pharmaceutical industry and materials science. Nevertheless, research in this area remains fragmented and requires a comprehensive review.

This review aims to systematically analyse and summarise the current data on adamantane biotransformation by bacteria, focusing particularly on the mechanisms of regioselective hydroxylation and other types of functionalisation carried out by bacterial enzymes, especially cytochromes P450 and monooxygenases. This review will evaluate the effectiveness and potential of biocatalytic methods for selectively modifying adamantane and its derivatives, which is crucial for developing new functional materials and pharmaceuticals.

## 2. Methods for Literature Search

A comprehensive search of the literature on the biodegradation and biotransformation of adamantane was conducted to prepare this review. The following keywords were used as search terms: ‘adamantane’, ‘adamantane derivatives’, ‘diaminoids’, ‘biotransformation’, ‘regioselectivity’, ‘biocatalysis’, ‘microbial degradation’, ‘bacterial transformation’, ‘hydroxylation’, ‘cytochrome P450’, ‘stereoselectivity’ and ‘functionalisation’.

Publications were selected based on the following criteria:

(1) Relevance to the topic of biocatalytic transformation and selective modification of adamantane;
(2) The availability of experimental data on regioselectivity and biocatalysis mechanisms;
(3) Inclusion of both classical and modern experimental and review studies, given the limited amount of work in this field.
(4) Emphasis was placed on enzymatic systems (in particular cytochromes P450 and monooxygenases) and bacterial strains (*Pseudomonas* spp.).
(5) Inclusion of data on the synthetic chemistry of adamantanes and catalytic systems for comprehensive coverage of the material.

The search was conducted in major scientific databases and with major publishers, including PubMed, CrossRef, Springer and Wiley, as well as on specialised platforms dedicated to petrochemistry and biocatalysis.

## 3. Structure and Properties of Adamantane

### 3.1. Chemical Structure of Adamantane

**Figure 1:**
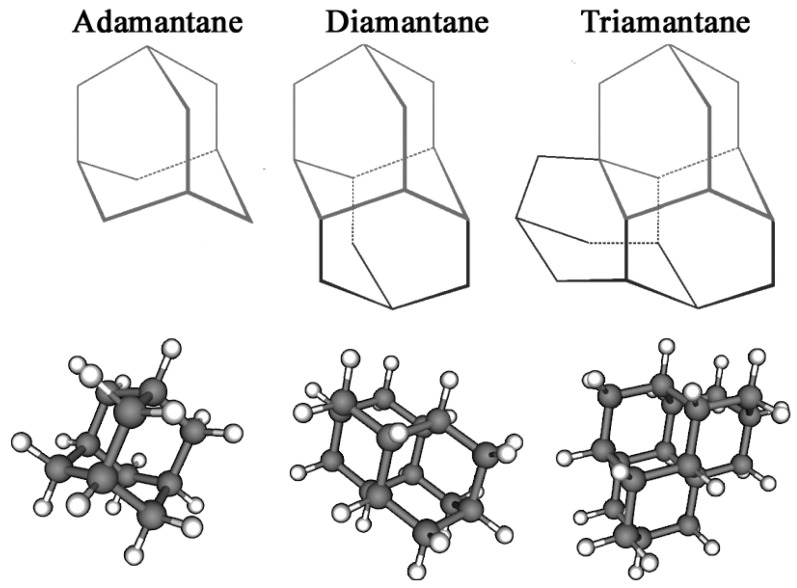
Chemical structure of diamondoids.

Adamantanes are a class of condensed, carbocyclic alkanes with a three-dimensional, polycyclic structure in which the carbon atoms are in an sp^3^ hybridisation state. These adamantane compounds are considered nanoscale fragments of the diamond lattice and are characterised by high symmetry and a unique set of physicochemical properties. The simplest representative of almasoids is adamantane (C_10_H_16_) (Figure 1), which has Td group symmetry. It is characterised by the presence of two types of hydrogen atom position in C–H bonds: four equivalent tertiary bridge positions and six methylene segments forming a tricyclic cell-type framework. Just as diamond is a hard, mechanically strong allotropic form of carbon, diamondoids are condensed hydrocarbon structures with high thermodynamic stability whose general empirical formula is C_4n+6_H_4n+12_. This series of compounds includes molecules with clearly defined geometries, such as diamantane and triamantane (see Figure 1), as well as less regular nanoscale forms known as ‘nanodiamonds’, which have even been discovered in interstellar space [20].

Adamantane is a colourless, crystalline substance that smells like camphor. It is slightly soluble in water, but dissolves easily in non-polar solvents. Its melting point is 269 °C and its boiling point is 194.5 °C. This is a very high boiling point for such a low-molecular-weight hydrocarbon, due to the molecule’s high symmetry. However, introducing alkyl groups into the adamantane nucleus breaks the symmetry and causes a sharp drop in the melting point (for example, the melting points of 1-methyladamantane and 1-ethyladamantane are 103 °C and −58 °C, respectively) [21].

Almazoids of all sizes are still being studied for their unique physical and spectroscopic properties, and for their potential applications in medicine and materials science [9].

Thanks to its unique symmetrical structure, high thermodynamic stability and unusual physicochemical properties, adamantane serves as a model compound for the study of diamondoids.

### 3.2. Biological Activity and Applications of Adamantane Derivatives

Due to its unique structure, adamantane possesses a variety of useful chemical and physical properties. It is a transparent, rigid substance with high thermal stability and pronounced hydrophobic and lubricating properties, which are due to the high density of C-H bonds and the weak intermolecular interactions that are characteristic of the adamantane structure.

Adamantane and its derivatives are widely used in the development of various pharmaceutical products [22].

It is believed that the biological effects of adamantane derivatives are largely due to the presence of a bulky, highly lipophilic backbone. The lipophilicity of the adamantane core determines the ability of its substituted derivatives to interact with biological membranes containing a lipid layer, as well as with hydrophobic protein fragments, including those that form part of receptor structures. It is known that adamantane derivatives can stabilise biological membranes [23].

For example, the antiviral mechanism of action of adamantanes in the treatment of influenza A is based on their interaction with the M2 transmembrane channel, which is a proton-selective ion channel formed by the M2 protein that transports protons across the lipid membrane of the viral envelope [24]. Additionally, adamantane derivatives affect the process of viral endocytosis, which is critical for penetration into the host cell and is accompanied by pH shifts. Their ability to increase the pH of endosomes prevents the formation of the infectious cycle in influenza and coronaviruses [25,26].

In addition to their direct antiviral action, adamantane derivatives exhibit immunomodulatory properties. They can reduce the severity of inflammatory processes by modulating microglial activity, thereby exerting an immunostimulatory effect. These effects have been confirmed in studies involving both animal models of diabetes with cognitive impairment and cell cultures, in which the inhibition of inflammatory activity in microglia has been observed [27,28,29].

An important area of medical application is neurology. The biological activity of adamantane and its derivatives in the treatment of Parkinson’s disease is associated with their ability to block N-methyl-D-aspartate (NMDA) receptor channels. These channels play a key role in excitatory neurotransmission in the central nervous system. It is this activity that explains the neuroprotective effects of drugs in this group [30].

Table 1 shows the main adamantane derivatives, their pharmacological effects, and their clinical applications, as confirmed by recent research.

Thanks to their unique combination of physicochemical properties and high structural adaptability, adamantane and its derivatives are important compounds in modern pharmaceutical chemistry and medicine, used to solve a wide range of problems.

## 4. Transformation of Adamantane and Its Derivatives

### 4.1. General Principles of Hydroxylation of C–H Bonds in Adamantane

The selective hydroxylation of aliphatic bonds is one of the most challenging and pressing issues in modern organic synthesis [54].

In nature, this type of reaction occurs with the help of enzyme systems such as cytochromes P450 and heme- and thiolate-containing monooxygenases. These enzymes can selectively functionalise energetically inert aliphatic C–H bonds by forming reactive high-valent iron oxo complexes. These complexes then mediate oxygen transfer and hydrogen atom cleavage, leading to the formation of oxygen-containing products during the catalytic cycle [55]. Table S1 (Supplementary Materials) summarises the comparative characteristics of various cytochrome P450 enzymes involved in the biotransformation of adamantane and camphor derivatives, highlighting their sources, substrates, products, regioselectivity patterns, and required cofactors.

An important feature of the natural enzymes responsible for the hydroxylation of adamantane compounds is their high regio- and stereoselectivity. This is due to the enzymes’ specific structure and binding mechanisms in the active centre. Bacterial monooxygenases (e.g., cytochromes P450) in particular ensure the directed introduction of a hydroxyl group by forming a transition complex with the substrate. The orientation of the molecule within the enzyme’s active site is then determined by weak electrostatic and hydrophobic interactions. X-ray structural and biochemical studies demonstrate that the size, shape and polarity of the binding region determine how the substrate is ‘locked’ into the optimal position for selective oxidation. This results in the preferential hydroxylation of the tertiary carbon atoms of the adamantane skeleton. These molecular mechanisms underpin the unique specificity of natural biocatalysts, explaining their superiority over traditional chemical oxidants [56].

In living systems, the oxidation of aliphatic C–H bonds is crucial for metabolic processes. It determines the biosynthesis of complex metabolites, such as hormones, antibiotics, neurotoxins and neurotransmitters. It is also involved in the detoxification of xenobiotics and the initiation of signalling pathways [57]. Natural enzymes exhibit high regio-, chemo- and stereoselectivity, even when multiple non-equivalent C–H bonds or other reactive functional groups are present, due to the active centre’s ability to exert specific stereochemical control over the substrate through a network of weak interactions. This enables specific carbon atoms in complex molecules to be modified in a targeted manner.

For cytochrome P450 and haem catalysts, adamantane exhibits regioselectivity of up to 48:1. This indicates that the catalyst causes a highly selective and efficient catalytic reaction [58].

Pharmacological preparations based on adamantane are oxidised in animals’ bodies with the help of the cytochrome P450 enzyme complex in the liver, forming mono- and dihydroxy derivatives.

### 4.2. Features of Bacterial Transformation of Adamantane

**Figure 2:**
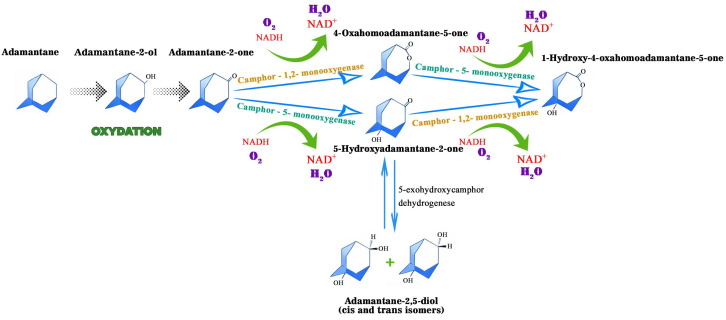
Proposed aerobic pathways for the bacterial oxidation of adamantane.

Diamond-like hydrocarbons are considered to be more resistant to microbial action than other petroleum components, such as n-alkanes. The microbial metabolism of adamantane and its derivatives is a poorly studied area, as evidenced by the limited number of publications on this topic. Research has particularly focused on the transformation of adamantane-series hydrocarbons under microbial action, but the mechanistic aspects of these transformations have only been studied fragmentarily so far. Nevertheless, microbial hydroxylation, characterised by high regioselectivity and stereoselectivity towards inactive alicyclic and heterocyclic carbon atoms, is a promising method for synthesising target organic compounds with potential practical applications [59]. An early study reported that cytochrome P450cam monooxygenase from *Pseudomonas putida* selectively oxidises adamantane to 1-adamantanol [19]. Its catalytic activity was moderately high, at 43 mol/mol P-450cam/min., a value comparable to the hydroxylation activity of the natural substrate camphor, which is 60 mol/mol P-450cam/min. In their study, Mitsukura et al. [59] investigated a strain of Streptomyces griseoplanus that exhibited high regional selectivity. After 72 h of cultivation in the presence of 3% (*v*/*v*) Tween 60, the strain formed 1-adamantanol from adamantane with a yield of 32%. The hydroxylation activity was significantly suppressed by a P450 inhibitor such as 1-aminobenzotriazole or menadione at a concentration of 0.5 mM. The authors suggested that the P450 oxidation system may be involved in this hydroxylation reaction. The study [60] showed that almazoids undergo biodegradation that is both selective and stepwise. They proposed a scheme of the presumed aerobic biotransformation pathways of adamantane degradation, reflecting the main stages and enzymatic mechanisms of its conversion in the presence of molecular oxygen (Figure 2). These pathways are primarily based on pathways found in camphor-degrading bacteria, like *Pseudomonas putida*.

According to the scheme of aerobic biotransformation of adamantane, the main metabolites formed when this compound is oxidised by Gram-negative saprotrophic bacteria such as *Pseudomonas putida* are 4-oxoadamantane-5-one and 5-hydroxyadamantane-2-one. These metabolites are produced by the action of camphor-1,2-monooxygenase and camphor-5-monooxygenase enzymes. These results emphasise the significant role of the enzyme systems involved in camphor degradation in transforming adamantane structures.

In his dissertation, A. V. Slepenkin [61] investigated the microbiological transformation of adamantane and alkyladamantanes using Pseudomonas aeruginosa BS315 cells that were grown using camphor as their sole source of carbon and energy. The study revealed that adamantane and adamantane-2-one undergo hydroxylation at tertiary carbon atoms in the presence of a purified preparation of cytochrome P450cam monooxygenase, which is encoded by the CAM plasmid (the camphor biodegradation plasmid) [19]. The *P. aeruginosa* BS315 strain used in the study contained three biodegradation plasmids (pBS, CAM and OCT (pBS250)) and was only able to transform adamantane after preliminary cultivation on camphor [61]. This suggests that the enzymatic systems responsible for degrading polyaromatic and linear aliphatic hydrocarbons are unable to participate in the biological transformation of adamantane and its derivatives. Analysis of the transformation products revealed the formation of adamantane-1-ol and adamantane-1,3-diol, as determined by chromatography-mass spectrometry. Hydroxylation of adamantane and its derivatives occurs exclusively on the tertiary carbon atoms of the molecular structure, leaving the secondary positions and alkyl substituents unaffected. A. V. Slepenkin’s [61] proposed metabolic pathway includes two sequential monohydroxylation stages of adamantane, where the monooxygenated derivatives serve as substrates for further oxidation.

Studies have shown that the oxidation of the adamantane nucleus occurs with high regiospecificity, suggesting that the hydroxylation of adamantane is likely associated with the activity of cytochrome P450cam-methylene monooxygenase (camphor-5-monooxygenase). Production of this enzyme complex is induced when bacteria are cultivated in a medium where camphor is the sole source of carbon and energy. As previously established, camphor-5-monooxygenase functions as a multi-enzyme ensemble in the bacterial system, including the thioredoxin reductase, thioredoxin and cytochrome P450cam pathways [62]. The expression of the corresponding operon is initiated not only by D-camphor, but also by several of its analogues and degradation products, such as adamantane, 2-adamantanol, 5-exo-hydroxycamphor, D-3-bromocamphor and 2,5-diketocamphane.

Due to their rigid, highly symmetrical structure, which is reminiscent of a diamond crystal lattice, adamantanoids demonstrate a uniquely high level of thermal stability for saturated hydrocarbons at a relatively low molecular weight, as well as notable resistance to biotransformation. Currently, there is no data confirming the ability of microorganisms to use adamantane or its derivatives exclusively as sources of carbon and energy.

### 4.3. Bacterial Transformation of Adamantane Derivatives

Research into identifying the intermediate products, pathways and molecular mechanisms of the microbial transformation of adamantane and its derivatives has significant potential for the production of new biologically active compounds in this class using microbiological methods. Adamantane is usually found in natural crude oil together with small amounts of its alkylated derivatives, such as 2-methyladamantane, 1-ethyladamantane and, possibly, 1-methyladamantane and 1,3-dimethyladamantane.

Hydroxylated adamantane derivatives are widely used as functional materials. For instance, acrylic esters of 1-adamantanol and 1,3-adamantanediol are in high demand for creating photoresistive coatings. The high selectivity of the hydroxylation process is particularly important when obtaining such compounds, in terms of both the chemical mechanism and the location at which the hydroxyl group is introduced into the adamantane structure [2].

The study by Mitsukura et al. [56] investigated the ability of the SA8 strain of *Streptomyces* sp. to selectively hydroxylate 1-adamantanol. The reaction yielded 1,3- and 1,4-adamantandiol. During this process, 2.3 g/L of 1,3-adamantanediol was formed at a conversion rate of 69%, while the by-product 1,4-adamantanediol accounted for about 15%. The study found that cells only exhibit hydroxylating activity when grown in the presence of inducing substrates. Various cycloalkanes were added to the culture medium to induce hydroxylating activity, including adamantane, 1-adamantanol, cyclohexane, cyclohexanol, cyclooctane, decalin, decalinol and (±)-camphor. 1-Adamantanol exhibited the greatest inducing ability. These results suggest that there is specialised regulation of the enzymatic systems that ensures the selective hydroxylation of adamantane derivatives.

Another study [1] showed that the susceptibility of diamond-like components to biological degradation is largely determined by the extent to which they are alkylated and by the number of cyclic fragments in the molecule. It has been established that compounds containing more alkyl substituents and ring structures exhibit significantly greater resistance to microbial degradation than less branched molecules. The size and geometry of molecules have been found to play an important role in permeability through cell membranes and in the specificity of interaction with the active centres of microorganisms. This explains why the biotransformation rate of multi-ring and highly alkylated alkyloids is lower. However, the molecular mechanisms that determine the selectivity of the microbial degradation of these compounds remain unclear [1].

In the work of Ivanova, A.E. et al. [52], the aerobic degradation of alkyl-substituted adamantane derivatives—1-methyl-, 1,3-dimethyl- and 1,3,5-trimethyladamantane—by slow-growing acidophilic bacteria Mycobacterium sp. AGS10 under extremely acidic pH conditions (2.5). The degradation of 1-methyladamantane as an individual compound was 54% over 20 days, whereas for 1,3-dimethyladamantane it was 98%. Data on the biodegradation of 1,3,5-trimethyladamantane are not available. In a gas condensate mixture with an increased content of 1-methyladamantane, the consumption rates were approximately 55%, 58%, and 64% for 1-methyladamantane, 1,3-dimethyladamantane, and 1,3,5-trimethyladamantane, respectively. In the original gas condensate with a complex composition, the degradation rates were 77% for 1-methyladamantane, 75% for 1,3-dimethyladamantane and 99% for 1,3,5-trimethyladamantane over 60 days.

As noted in published works, most known oil-degrading bacteria do not typically use adamantane structures as their sole source of carbon. One mechanism by which microbes act on stable compounds is considered to be concomitant oxidation (co-metabolism), whereby hard-to-process compounds are degraded in the presence of more easily degradable substrates. Experiments showed that, when present together in a culture, the microbial degradation of methyl- and dimethyladamantane was 55% and 58%, respectively. Notably, when these compounds were used individually, the consumption rate of liquid 1,3-dimethyladamantane at 30 °C was almost twice that of solid 1-methyladamantane [51].

A study by S. A. Selifonov [63] demonstrated that intact *Pseudomonas putida* ATCC 17453 cells, which carry a camphor catabolic plasmid and have been pre-grown on camphor, can effectively oxidise the adamantane monoketone derivative to produce saturated oxygen-containing compounds. The authors state that the identified products indicate the occurrence of both biocatalysed Beyer–Williger reactions and hydroxylation reactions. Consequently, metabolites such as 4-oxahomoadamantane-5-one, 5-hydroxyadamantane-2-one and 1-hydroxy-4-oxahomoadamantane-5-one were identified. Additionally, syn- and anti-1,4-dihydroxyadamantanes and bicyclo [3.3.1]nonan-3-ol were formed during secondary transformations.

Folwell B.D. et al. [64] studied the aerobic biotransformation of the diamondoid carboxylic acids adamantane-1-carboxylic acid and 3-ethyl adamantane carboxylic acid by microbial communities isolated from the various aquatic ecological niches of the Mildred Lake tailings pond. Over a period of 33 days, these acids underwent microbial transformation, resulting in a simultaneous decrease in toxicity. Two presumed metabolites were identified: 2-hydroxyadamantane-1-carboxylic acid (derived from adamantane-1-carboxylic acid) and 3-ethyladamantane-2-ol (derived from 3-ethyl adamantane carboxylic acid). The authors hypothesise that certain microorganisms within the enriched, isolated communities can use alkyloxy naphthenic acids as their sole sources of carbon and energy for growth. Evidence for this includes the absence of metabolite accumulation proportional to substrate depletion, the absence of new peaks (suggesting further degradation) and the dominance of *Pseudomonas* by day 33, as well as the fact that alkyloxy acids were the only available source of carbon and energy. In addition to *Pseudomonas*, active taxa included *Cellvibrio*, *Bacillus*, *Devosia* and *Brevundimonas*.

In a study by Mitsukura et al. [59], the ability of soil microorganisms to hydroxylate 1,3-adamantandiol was investigated. The study found that the actinobacterium *Kitasatospora* sp. GF12 effectively synthesises 1,3,5-adamantatriol. Two possible biochemical pathways were identified: direct regioselective hydroxylation of 1,3-adamantandiol, or two-step hydroxylation of 1-adamantanol, which also leads to the target triol.

Table 2 provides examples of the microbial transformation of various adamantane derivatives, as documented in the literature. This table indicates the bacterial strains or microbial communities used, the target substrates, the products obtained and the corresponding scientific sources.

The examples presented thus demonstrate the high potential of bacterial and microbial systems for the selective transformation of adamantane derivatives into unique products.

### 4.4. Functionalisation of Adamantane: Chemical and Biocatalytic Strategies

The functionalisation of adamantane is a key area of modern organic chemistry. Obtaining its derivatives with specified properties significantly expands the possibilities for the practical application of diamondoids in various fields of science and technology.

Table 3 outlines the primary approaches to adamantane functionalisation employed in chemical and biocatalytic synthesis. The key advantages and limitations of each method are summarised, and data on target products and reaction yields are provided based on scientific research data. Particular attention is paid to biocatalytic methods using cytochrome P450 enzymes and microorganisms, which ensure the processes are highly regioselective and environmentally friendly. This analytical material enables the effectiveness of various methods of selective adamantane modification to be compared and their potential for targeted production of functional derivatives to be assessed.

Selectivity increases significantly when moving from traditional chemical methods to biocatalytic and microbial methods. The highest values (>80–90%) are reached in enzymatic and whole-cell transformations due to the precise control of hydroxylation sites. However, yields generally remain moderate across all methods, ranging from low values (10–35%) in biotransformations to higher levels (up to 80%) in catalytic chemical reactions.

The functionalisation of adamantane using chemical methods, including traditional radical substitution and catalytic C–H bond functionalisation with transition metals, is significantly limited. Traditional radical reactions are characterised by low selectivity, harsh reaction conditions and the formation of complex product mixtures with undefined yields. Although catalytic methods offer better selectivity and more controlled conditions, they require complex and expensive catalysts that are sensitive to water and oxygen. Furthermore, chemical oxidative hydroxylation using peroxides and other oxidants is often characterised by low selectivity, side reactions and the requirement for high temperatures, which limits its efficiency and practical application.

Biocatalytic methods often face limitations that hinder their widespread application. For instance, cytochrome P450 enzymes, such as P450cam, exhibit low catalytic activity towards adamantane as it is not their native substrate. Consequently, substrate binding is poor, and electron transfer is inefficient, resulting in reduced reaction rates and product yields. Furthermore, the activation of these enzymes necessitates the presence of specific inducers, such as camphor, as well as intricate multi-component systems comprising electron carriers and auxiliary proteins. This significantly complicates the scaling up of biocatalytic processes, increasing their cost due to the need for additional reagents and control of reaction conditions. These factors emphasise the importance of developing and implementing bioengineering approaches that improve enzyme specificity and stability and simplify application systems.

Thus, the unique advantages and limitations of modern methods of adamantane functionalisation can be exploited through their rational selection to enable the targeted production of derivatives with specified properties.

## 5. Current Strategies and Tools for Potential Bioengineering Approaches to the Selective Biotransformation of Adamantanes

Modern bioengineering strategies can be employed to enhance the yield and specificity of enzymes involved in the biotransformation of adamantane and its derivatives. For instance, directed evolution methods can be employed to obtain enzymes with enhanced activity through random or targeted mutagenesis, followed by high-throughput screening of mutant variants [66,67]. Such approaches have already been shown to be effective in enhancing the oxidative activity of cytochromes P450 in the hydroxylation of complex alicyclic substrates [19] and are likely to be applicable to adamantane. Alongside random mutagenesis, rational design techniques are employed that are based on the analysis of the enzyme’s three-dimensional structure and the molecular docking of the substrate in order to specifically modify the amino acid environment of the active site [68,69]. The creation of hybrid enzyme complexes with increased stability and selectivity through the modification of coenzyme-binding domains or the inclusion of additional cooperative stabilisation sites is considered successful [70,71]. Modern plasmid systems enable the expression of optimised enzyme variants in heterologous bacterial or yeast cells to increase product yield [71,72,73]. Protocols for co-expression of accompanying redox partners (e.g., putidaredoxin and putidaredoxin reductase for bacterial cytochromes) have been developed, which significantly increases the efficiency of the complete catalytic cycle [74,75]. Metagenomic screening and synthetic biology can be used to construct entirely new catalytic pathways based on modular assembly principles [75]. Therefore, creating biocatalytic ensembles based on coupled enzyme cascades that allow for the stepwise selective functionalisation of adamantane with minimal product loss may also be promising.

## 6. Conclusions

Adamantane is a unique tricyclic alkane with a rigid, three-dimensional, diamond-like structure. It possesses high chemical stability and pronounced hydrophobicity and lipophilicity. These properties determine its use in medicine (in neuroprotective, antiviral and antitumour drugs), materials science (in three-dimensional framework structures and nanomaterials) and pharmaceutical drug delivery systems. The most notable adamantane derivatives are amantadine, memantine and remantadine, which are recognised as effective treatments for viral infections and neurodegenerative diseases.

The high resistance of adamantane to chemical and microbial degradation has long complicated the production of selectively modified derivatives. Traditional chemical methods of hydroxylation and functionalisation were limited in terms of selectivity and conditions, stimulating the search for biocatalytic solutions. Biotransformation of adamantane by microorganisms, particularly via the action of cytochrome P450 and related monooxygenases, exhibits high regiospecificity. This process selectively hydroxylates tertiary C-H bonds, forming mono- and dihydroxy derivatives that have potential applications.

Bacteria belonging to the *Pseudomonas* genus (e.g., *P. putida* ATCC 17453 (CAM) and *P. aeruginosa* BS315 strains), which possess a camphor biodegradation plasmid, as well as actinobacterial strains belonging to the *Streptomyces* and *Kitasatospora* genera, are capable of this process. The mechanisms of enzymatic hydroxylation are associated with multicomponent enzyme complexes (e.g., camphor-5-monooxygenase), which are induced by camphor or similar substrates. These processes provide relatively mild conditions and high selectivity of modification for the environment, which is invaluable for many chemical catalytic systems.

Modern research shows that the microbial transformation of adamantane and its derivatives mainly involves the oxidation of the tertiary carbon atoms in the nucleus to form stable hydroxy products. However, most known strains cannot utilise adamantane as their sole source of carbon and energy. The use of specialised microorganisms, including extremophiles, or co-oxidation with other substrates, can significantly expand the possibilities for biotransformation.

The latest data confirm the importance of microbial communities, including the genera *Pseudomonas*, *Cellvibrio*, *Bacillus*, *Devosia* and *Brevundimonas*. These communities are capable of transforming diamondoid carboxylic acids with reduced toxicity, opening up the potential use of these compounds in biotechnology. The pathways of the aerobic oxidation of adamantane have been studied, including the formation of the key metabolites 4-oxagomoadamantane-5-one and 5-hydroxyadamantane-2-one. This indicates a close connection between biotransformation and the enzyme systems involved in camphor degradation.

One promising approach is to induce and regulate enzymatic activity in microorganisms (e.g., by adding 1-adamantanol or other inducers) to improve the selectivity and yield of the desired derivatives. This is important for synthesising pharmaceuticals and functional materials. Biocatalytic methods offer the advantages of environmental friendliness, mild reaction conditions and high specificity. This is particularly important for the functionalisation of low-activity C-H bonds in adamantane. The field of bacterial adamantane transformation is expected to develop rapidly due to the integration of modern molecular genetic tools and metagenomic technologies. This will enable the identification and exploitation of previously unknown enzymes with unique specificity and stability. Applying directed evolution, optimising expression and stabilising biocatalytic complexes will increase the yield of target derivatives and expand the range of catalysed reactions. Studying enzyme gene regulation and searching for extremophilic strains capable of operating under a wide range of conditions is of particular importance.

Thus, integrating microbiology, enzymology, and organic synthesis enables us to solve the complex problem of the selective structural modification of adamantane and its derivatives. It also opens up prospects for further fundamental and applied research. Searching for new microorganisms with powerful enzyme systems for adamantane and its derivatives and developing a deep understanding of the mechanisms of enzymatic action and interaction with the substrate will increase regioselectivity and expand the possibilities of using biocatalysis to create innovative adamantane-based drugs and biomaterials.

## Figures and Tables

**Table 1:** Adamantane Derivatives and Their Applications.

No	Name of Adamantane Derivative	Application and Role	References
1	Amantadine 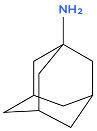	Antiviral (influenza A virus), anti-Parkinson’s agent	[31,32,33,34,35]
2	Memantine 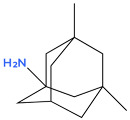	Treatment of neurodegenerative diseases (Alzheimer’s disease), neurotropic agent	[11,31]
3	Rimantadine (remantadine) 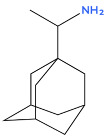	Antiviral (against influenza A virus), treatment of Parkinson’s disease	[32,36]
4	Tromantadine 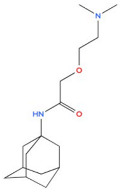	Analogue of amantadine, antiviral action	[37]
5	Vildagliptin 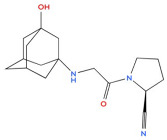	Hypoglycaemic agent for the treatment of type 2 diabetes mellitus, antidiabetic activity	[38,39,40]
6	Bromantane 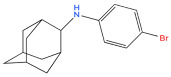	Immunotropic, antiviral activity, adaptogen, psychostimulant, actoprotector	[41,42,43]
7	Nitromemantine (nitrosynapsin, YQW-036 or EM-036) 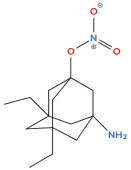	Treatment of neurodegenerative diseases, neuroprotective effect	[44]
8	Saxagliptin 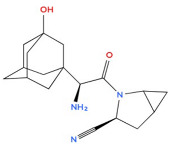	Used in the treatment of diabetes mellitus, improvement of glycaemic control	[40,45]
9	Gludantan 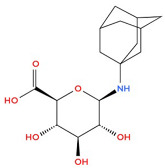	Anti-Parkinson’s agent, antidepressant, antiviral	[46]
10	Adapromine 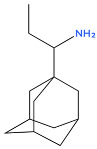	An anti-Parkinson’s disease, antidepressant and psychostimulant agent with antiviral properties	[47,48]
11	Diamantane 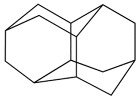	Vector for delivering anti-cancer drugs	[49]
12	Kemanatan 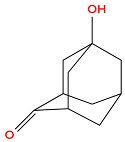	Immune-stimulating effect	[50,51]
13	Hemanthan 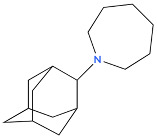	Anti-Parkinson’s agent	[52]
14	Diadonium (dithosylate bis[di(2-(adamantyl dimethylammonio)ethyl)succinate]) 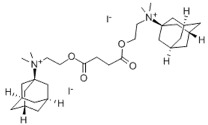	Short-acting non-depolarising muscle relaxant	[53]
15	Chlodanthan 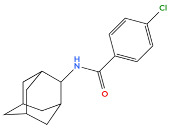	Adaptogen, immunostimulant	[42]

**Table 2:** Known examples of adamantane derivative transformation by bacterial strains or microbial communities.

No	Microorganism	Substrate	Transformation Products	References
1	*Streptomyces* sp. SA8	1-adamantanol	1,3-adamantandiol and 1,4-adamantandiol	[56]
2	Microbial communities from various aquatic ecological niches of the Mildred Lake Tail Basin	Adamantane carboxylic acids: adamantane-1-carboxylic acid, 3-ethyl adamantane carboxylic acid	2-hydroxyadamantane-1-carboxylic acid, 3-ethyladamantane-2-ol	[64]
3	*Kitasatospora* sp. GF12	1,3-Adamantandiol	1,3,5-Adamantatriol	[65]
4	*Mycobacterium* AGS10	1-Methyl-, 1,3-dimethyl- and 1,3,5-trimethyladamantanes	Partial consumption of ~55–58% of methyl- and dimethyladamantanes (metabolites not identified)	[52]
5	*Pseudomonas putida* ATCC17453(CAM)	Monoketone derivative of adamantane (adamantanone)	4-oxahomoadamantane-5-one; 5-hydroxyadamantane-2-one; 1-hydroxy-4-oxa-homoadamantane-5-one; syn- and anti-1,4-dihydroxyadamantanes	[63]

**Table 3:** Comparison of methods for the functionalisation of adamantane.

No	Method Name	Advantages	Limitations	Products/Outputs	Selectivity	Yield (Approx.)	References
1	Traditional chemical radical substitution of C–H	- High reactivity - Variety of modifications	- Low selectivity - Aggressive conditions - Product mixtures	Substituted adamantanes, often complex mixtures; exact yields not specified	Low (multiple C–H positions affected, poor control)	Variable, usually low (often <20%, product mixtures)	[9]
2	Catalytic functionalisation of C–H bonds using transition metals (e.g., Pd, Rh).	- Increased selectivity - Controlled conditions	- Complex catalysts required - Sensitivity to water and oxygen - Cost	Hydroxylation predominantly occurs at the tertiary C–H bond, with high regioselectivity.	High regioselectivity (tertiary C–H), typically >90%	Moderate to high (30–80% depending on substrate and catalyst)	[17]
3	Chemical oxidative hydroxylation using peroxides and other oxidants.	- Simple reagents - Obtaining oxygen-containing products	- Low selectivity - Side reactions - Often high temperatures culture	Mono- and dihydroxy derivatives of adamantane were obtained with moderate yields	Moderate (primary and secondary C–H, possible side reactions)	Moderate (20–60%)	[58]
4	Biocatalytic hydroxylation using cytochrome P450 (P450cam) and other monooxygenases	- Very high regioselectivity (up to 48:1). - Mild conditions and environmental friendliness	- Low catalytic activity - Need for induction - Susceptibility to inhibitors	1-Adamantanol (up to 32% yield), dihydroxy derivatives (1,3-adamantanediol, 1,4-adamantanediol, etc.).	Very high (up to 48:1 for tertiary vs. secondary C–H, e.g., P450cam)	Up to 32% for 1-adamantanole; other derivatives 5–20%	[19,59]
5	- Microbial transformation (Streptomyces, *Pseudomonas*, *Kitasatospora* strains, etc.).	- High selectivity and specificity - Mild conditions - Environmental friendliness	- Slow reaction rate - Scaling difficulties - Need to maintain	1-adamantanol; 1,3-adamantanediol; 1,4-adamantanediol; 1,3,5-adamantatriol; 4-oxagomoadamantane-5-one; 5-hydroxyadamantane-2-one, etc.).	High (typically >80% for target positions, strain-dependent)	Low to moderate (10–35% for main products)	[56,64,65]

## Data Availability

Data are available from the corresponding author upon reasonable request.

## Supplementary Materials

The following supporting information can be downloaded at: https://www.mdpi.com/article/10.3390/biology14101429/s1, Table S1. Cytochrome P450 Enzymes in Adamantane and Camphor Metabolism: Comparative Characteristics. References [76,77] are cited in the Supplementary Materials.
