# Supplementary material for: Bacterial Transformation of Adamantane and Its Derivatives: Regioselectivity and Biocatalytic Approaches
Source: Biology (Basel). 2025 Oct 17;14(10):1429. doi: 10.3390/biology14101429 (PMC12562002; doi:10.3390/biology14101429)
Supplement: Supplementary file 1 [file biology-14-01429-s001.zip › biology-3903050-supplementary.pdf]

# Supplementary Materials

## Bacterial Transformation of Adamantane and Its Derivatives: Regioselectivity and Biocatalytic Approaches

**Anastasia Ivanova * and Anna Vetrova**

Federal Research Center "Pushchino Scientific Center for Biological Research of the Russian Academy of Sciences", 142290 Pushchino, Russia; phdvetrova@gmail.com
* Correspondence: mrs.ivanova.a.a@gmail.com

**Table.S1.** Cytochrome P450 Enzymes in Adamantane and Camphor Metabolism: Comparative Characteristics

| Enzyme Name/Class | Source | Substrate | Product | Observed Regioselectivity | Cofactors | Reference |
|---|---|---|---|---|---|---|
| P–450cam | *Pseudomonas putida* | Adamantane | 1-adamantanol | 1-position (100%) | NADH, putidaredoxin, putidaredoxin reductase | [19] |
| P–450LM2 | Rabbit liver | Adamantane | 1-adamantanol,<br>2-adamantanol | 1-position (91%),<br>2-position (9%) | NADPH, NADP+, isocitrate, $MGCL_2$, dilauroylglyceryl-3-phosphorylcholine | [19] |
| Cytochrome P-450 (presumably) | *Streptomyces griseoplanus* AC122 | Adamantane | 1-adamantanol,<br>2-adamantanol | 1-position (89%),<br>2nd position (11%) | Not explicitly specified, but the P-450 system is required (inhibited by 1-aminobenzotriazole | [59] |

| Enzyme Name/Class | Source | Substrate | Product | Observed Regioselectivity | Cofactors | Reference |
|---|---|---|---|---|---|---|
| | | | | | imedione) | |
| CYP109B1 (cytochrome P450) | *Escherichia coli* BL21(DE3) with plasmid pET-109B1-CamA-CamB | N-(2-adamantyl)-benzamide | N-(5-hydroxy-2-adamantyl)-benzamide | 5-position of adamantane | NADPH, recovery system (CamA-CamB) | [76] |
| CYP109FK (fusion protein) | *Escherichia coli* BL21(DE3) with modified plasmid | N-(2-adamantyl)-benzamide | N-(5-hydroxy-2-adamantyl)-benzamide | 5-position of adamantane | integrated reduction domain | [76] |
| CYP109FK mutants (I77F, I77W, M105I, etc.) | *Escherichia coli* BL21(DE3) with modified plasmid | N-(2-adamantyl)-benzamide | N-(5-hydroxy-2-adamantyl)-benzamide | 5-position of adamantane | integrated reduction domain | [76] |
| P450cam (CYP101A1) | *Pseudomonas putida* | Camphor | 5-exo-hydroxycamphora | no data is provided | NADH, putidaredoxin (Pdx), putidaredoxin reductase (Pdr) | [77] |
| CYP101D1 | *Novosphingobium aromaticivorans,* although the protein itself was obtained by heterologous expression in *E. coli.* | Camphor | 5-exo-hydroxycamphora | no data is provided | no data is provided | [77] |
| P450cam mutants | *E. coli* (recombinant) | Camphor | 5-exo-hydroxycamphora | no data is provided | NADH, putidaredoxin (Pdx), putidaredoxin | [77] |

| Enzyme Name/Class | Source | Substrate | Product | Observed Regioselectivity | Cofactors | Reference |
|---|---|---|---|---|---|---|
| (L358A, K178G, L358A/K178G, L358A/K178G/ D182N) | | | | | reductase (Pdr) | |
| P450cam (CYP101A1) | *Pseudomonas putida* | Camphor | 5-exo-hydroxycamphora | no data is provided | NADH, putidaredoxin (Pdx), putidaredoxin reductase (Pdr) | [77] |
| Цитохром P-450cam (CYP101) | *Pseudomonas putida PpG1* | D-camphor | 5-exo-hydroxycamphora | no data is provided | NADH, putidaredoxin (CamB), NADH-putidaredoxin reductase (CamA) | [62] |
| 5-exo-hydroxycamphor dehydrogenase (FdeH) | *Pseudomonas putida PpG1* | 5-exo-hydroxycamphora | no data is provided | no data is provided | no data is provided | [62] |
| Cytochrome P-450cam (CYP101) | *Pseudomonas putida* PpG1 (CAM) | Adamantane | 1-adamantanol | 1-position | NADH, putidaredoxin (CamB), NADH-putidaredoxin reductase (CamA) | [63] |

| Enzyme Name/Class | Source | Substrate | Product | Observed Regioselectivity | Cofactors | Reference |
|---|---|---|---|---|---|---|
| Cytochrome P-450cam (CYP101) | *Pseudomonas putida* PpG1 (CAM) | Adamantanon | 5-hydroxyadamanthanone-2 | 5-position | NADH, putidaredoxin (CamB), NADH-putidaredoxin reductase (CamA) | [63] |
